# Impact of whole genome sequencing on the investigation of food-borne outbreaks of Shiga toxin-producing *Escherichia coli* serogroup O157:H7, England, 2013 to 2017

**DOI:** 10.2807/1560-7917.ES.2019.24.4.1800346

**Published:** 2019-01-24

**Authors:** Claire Jenkins, Timothy J Dallman, Kathie A Grant

**Affiliations:** 1 National Infection Service, Public Health England, United Kingdom

**Keywords:** food-borne infections, Escherichia coli O157, gastrointestinal disease, molecular methods, United Kingdom, zoonotic infections, bacterial infections, gastrointestinal disease, enterohaemorrhagic E. coli, EHEC, laboratory surveillance, molecular methods, typing

## Abstract

We aim to provide insight and guidance on the utility of whole genome sequencing (WGS) data for investigating food-borne outbreaks of Shiga toxin-producing *Escherichia coli* (STEC) O157:H7 in England between 2013 and 2017. Analysis of WGS data delivered an unprecedented level of strain discrimination when compared with multilocus variable number tandem repeat analysis. The robustness of the WGS method ensured confidence in the microbiological identification of linked cases, even when epidemiological links were obscured. There was evidence that phylogeny derived from WGS data can be used to trace the geographical origin of an isolate. Further analysis of the phylogenetic data provided insight on the evolutionary context of emerging pathogenic strains. Publically available WGS data linked to the clinical, epidemiological and environmental context of the sequenced strain has improved trace back investigations during outbreaks. Expanding the use of WGS-based typing analysis globally will ensure the rapid implementation of interventions to protect public health, inform risk assessment and facilitate the management of national and international food-borne outbreaks of STEC O157:H7.

## Background

In the 1980s, the emergence of Shiga toxin-producing *E. coli* (STEC) O157:H7, and the increasing number of outbreaks of gastrointestinal disease and haemolytic uraemic syndrome (HUS) associated with this serotype, stimulated the development of sub-typing methods that provided a higher level of strain discrimination than serotyping [1,2]. Phage typing was adopted by Public Health England (PHE) and is still used today [3]. In the1990s and 2000s, Pulsed field gel electrophoresis (PFGE) and multilocus variable number tandem repeat (VNTR) analysis (MLVA) respectively, were used reactively in outbreaks that had already been identified by epidemiological links, although from 2012 prospective typing using MLVA was also in use [4,5]. PHE implemented whole genome sequencing (WGS) as the molecular typing method of choice for all isolates of STEC O157:H7 in June 2015 [6]. The aim of this perspective is to summarise the evaluation and share experiences on the utility of whole genome sequencing (WGS) data for investigating food-borne outbreaks of STEC O157:H7 in England and discuss the impact of this approach on informing risk assessment and risk management of this clinically important food-borne pathogen.

## Preliminary evaluation studies and comparisons with multilocus variable number tandem repeat analysis

To assess the epidemiological relevance of genetic similarity between genomes a retrospective comparison was performed in 2014. Randomly selected isolates (n = 572) from the bacterial strain collection archive held at PHE were sequenced in order to assess the applicability of a common source single nucleotide polymorphism (SNP) variation threshold for outbreak detection, based on temporal and epidemiological linkages between isolates [6]. This study showed that, at the core genome level, isolates of STEC O157:H7 greater than five SNPs different were less likely to be part of the same temporally linked outbreak than those less than five SNPs different. During these preliminary investigations, previously unidentified clusters of isolates that fell within five SNPs of each other were detected; however, all but two of these clusters were too small to support meaningful epidemiological analysis. Following further epidemiological investigations, one of the two larger clusters was ultimately linked to consumption of contaminated salad leaves and the other was associated with exposure to animals at a national park [6]. Subsequent studies revealed that deeper phylogenetic relationships may provide epidemiologically useful information or associations [7,8] (Table 1).

**Table 1:** Use of whole genome sequencing to inform investigations of outbreaks of STEC O157:H7 and context, England 2013–2017

Reference	Description	Benefits of the WGS approach and context
Butcher et al. 2016 [7]	Outbreak of STEC O157:H7 PT21/28 associated with raw drinking milk in the south-west of England, 2014	• Robust, high level strain discrimination compared with traditional molecular typing methods • Forensic level typing for case ascertainment • Inferring the geographical origin of an outbreak strain from the phylogeny at the national level • Domestic source of outbreak strain • Evolutionary context of outbreak strains
Jenkins et al. 2015 [28]	Two national concurrent outbreaks of STEC O157:H7 PT2 associated with contaminated watercress, 2013	• Inferring the geographical origin of an outbreak strain from the phylogeny at the national and international level • Domestic source of outbreak strain • Non-domestic source of outbreak strain • Evolutionary context of outbreak strains
Mikhail et al. 2017 [8]	National outbreak of STEC O157:H7 PT8 associated with contaminated prepacked mixed leaf salad, 2015	• Inferring the geographical origin of an outbreak strain from the phylogeny at the national level • Domestic source of outbreak strain • Evolutionary context of outbreak strains
Byrne et al. 2016 [22]	Epidemiological and Microbiological Investigation of an Outbreak of Severe Disease from Shiga Toxin-Producing *Escherichia coli* O157 Infection Associated with Consumption of a Slaw Garnish	• Robust, high level strain discrimination compared with traditional molecular typing methods • Forensic level typing for case ascertainment • Domestic source of outbreak strain
Wilson et al. 2018 [24]	Outbreak of STEC O157:H7 PT21/28 associated with contaminated meat products at two butchers’ premises in the north-east of England, 2015	• Forensic level typing for case ascertainment • Domestic source of outbreak strain
Rowell et al. 2016 [25]	Outbreak of STEC O157 PT21/28 associated with a lamb-feeding event	• Robust, high level strain discrimination compared with traditional molecular typing methods • Forensic level typing for case ascertainment • Domestic source of outbreak strain
Underwood et al. 2014 [27]	Outbreak of STEC O157:H7 at an open farm in the south-east of England, 2009	• Robust, high level strain discrimination compared with traditional molecular typing method • Domestic source of outbreak strain
Gobin et al. 2018 [31]	National outbreak of Shiga toxin producing *E. coli* O157: H7 linked to mixed salad leaves, 2016.	• Inferring the geographical origin of an outbreak strain from the phylogeny at the international level • Non-domestic source of outbreak strain • Evolutionary context of outbreak strains
Cowley et al. 2016 [32]	Two related sequential outbreaks of STEC O157:H7 PT8 and PT54 associated with the same restaurant, 2013	• Robust, high level strain discrimination compared with traditional molecular typing methods • Inferring the geographical origin of an outbreak strain from the phylogeny at the international level • Non-domestic source of outbreak strain • Evolutionary context of outbreak strains

STEC: Shiga toxin-producing *Escherichia coli*; WGS: whole genome sequencing.

## Methods applied for sequencing and typing STEC O157:H7

DNA from isolates of STEC O157:H7 was extracted on the QiaSymphony (Qiagen, Germany), and sequenced on the HiSeq 2500 platform (Illumina Inc, United States (US)) yielding paired-end reads of 100bp in length. High quality reads were mapped to the reference STEC O157:H7 strain, Sakai (GenBank accession BA000007), using Burrows-Wheeler Aligner – Maximum Exact Matching (BWA MEM) [9]. The sequence alignment map output from BWA were sorted and indexed to produce a binary alignment map (BAM) using Samtools [9]. Genome Analysis Toolkit (GATK2) was then used to create a variant call format (VCF) file from each of the BAMs, which were further parsed to extract only SNP positions of high quality (mapping quality (MQ) > 30, depth (DP) > 10, variant ratio > 0.9) [10,11].

Hierarchical single linkage clustering was performed on the pairwise SNP difference between all isolates at descending distance thresholds (Δ250, Δ100, Δ50, Δ25, Δ10, Δ5, Δ0). The result of the clustering is a SNP profile, or SNP address, that is used to describe the population structure based on clonal group membership, as indicated by the number at each level of the seven-number SNP address [12]. Shiga toxin (Stx) subtyping was performed as described elsewhere [13].

## Timely resolution and improved case ascertainment during outbreak investigations

Published studies comparing PFGE and MLVA, and WGS for typing STEC conclude that WGS is the superior technique [14-19]. Using a survival analysis, Dallman et al. [6] showed in a study published in 2015, that there was no significant temporal difference between MLVA and WGS SNP typing with respect to the time to identify a cluster, i.e. WGS was as sensitive as MLVA with respect to detecting an outbreak. However, when the time to cluster completion (the rate all cases of a cluster are clustered) from the initial cluster event (any two cases of a cluster are clustered) was reviewed, there was a significant speed increase in rate of completion of clusters with WGS when compared with MLVA. Other studies have also highlighted the considerable confidence WGS data affords in assigning ‘like’ vs ‘not-like’ status to two potentially linked bacteria [20].

**Figure 1:**
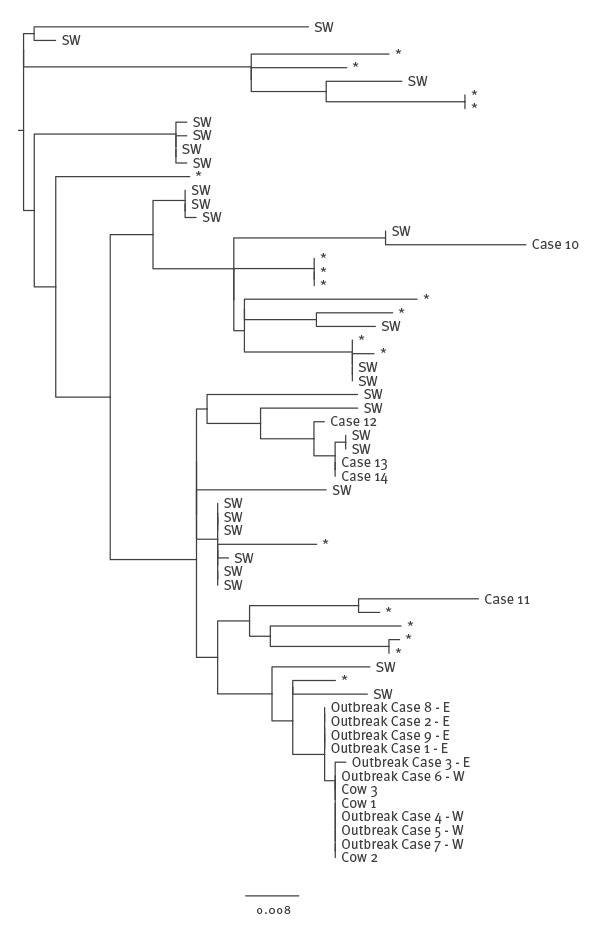
Phylogenetic relationship between isolates from human Shiga toxin-producing *Escherichia coli* O157:H7 PT21/28 cases linked to consumption of raw milk and cattle, and isolates from sporadic human clinical cases that fell within a 25 SNP cluster of the outbreak isolates, England, 2014

RDM: raw drinking milk; MLVA: multilocus variable number tandem repeat analysis; SNP: single nucleotide polymorphism; WGS: whole genome sequencing.

 This level of confidence in the microbiological typing data improves case ascertainment during outbreak investigations. In September 2014, the national enhanced STEC surveillance system [21] detected five cases associated with the consumption of raw drinking milk (RDM) produced at a farm in the south-west of England [7] (Table 1). Real time MLVA surveillance identified an additional nine isolates that appeared to be closely related to the outbreak strain by MLVA; there was uncertainty as to whether these additional cases were linked to the outbreak, as none of the cases reported RDM consumption on the STEC enhanced surveillance questionnaire (Figure 1, Cases 4–7, 10–14). Analysis of the WGS data revealed that four of these nine cases were part of the outbreak (Figure 1, Cases 4–7) and five were not associated with the outbreak (Figure 1, Cases 10–14). The nine cases were re-interviewed and asked questions about their consumption of dairy products, a search was also carried out for their names and postcodes on the distribution list supplied by the operations manager at the implicated farm. Subsequent epidemiological investigations provided evidence that the four cases, identified by WGS as being linked to the outbreak, had consumed RDM but initially failed to recall an accurate food history or were unaware that the milk was unpasteurised; no evidence of consumption of RDM was uncovered for the remaining five cases identified by MLVA only [7]

Cases 1, 2, 3, 8 and 9, initially identified by epidemiological links (reporting the consumption of RDM from the same farm), are designated ‘E’. Cases 4–7 initially identified by analysis of the WGS data (and subsequently found to have consumed RDM from the implicated farm), are designated ‘W’. Cases 10–14 were identified as potentially linked to the outbreak by MLVA, but were shown not to be directly linked by WGS and subsequent epidemiological investigations. Cases designated SW resided in the south-west of England, but did not report consumption of RDM.

It has been shown that epidemiological investigations are often confounded by poor patient recall of the food they consumed before onset of symptoms, particularly when the product is a side dish (e.g. salad leaves or raw vegetables) or an ingredient of the main dish (e.g. herbs or spices), so called ‘stealth vehicles’ [22,23]. The forensic-level microbiological typing provided by WGS can be used to generate a robust case definition for case ascertainment, even when the epidemiological links are obscured by poor patient recall of their history of food consumption [24,25].

## Inferring the geographical origin and/or potential animal reservoir of a food-borne outbreak strain

WGS data offers robust, high-level phylogenetic resolution and utilises quantifiable genetic markers that provide insight on the evolutionary context of an outbreak strain. Analysis of the data from the STEC O157:H7 dataset held at PHE showed that by exploring the context of the deeper phylogenetic relationship between isolates, the source of infection could be linked to specific geographical regions of the United Kingdom (UK).

For example, the farm implicated in the RDM outbreak in 2014 was located in the south-west of England [7] (Table 1). Even though none of the cases within the same 25 SNP cluster as the outbreak strain reported consumption of RDM on the STEC-enhanced surveillance questionnaire, epidemiological analysis showed that 23/33 (70%) of these cases were resident in the south-west of England or had travelled there within 7 days before the onset of illness (Figure 1). Spatial analysis of the geographical location of the presumed exposure of the STEC O157:H7 cases within this 25 SNP cluster revealed a highly significant cluster in the south-west of England region. Rates of infection with this strain were significantly lower in other parts of England. This analysis provided evidence that the source of infection for outbreaks and sporadic cases of STEC O157:H7 in the UK may be geographically restricted and that it may be possible to map the location of the source using an phylogenetic approach, thus providing an evidence base to direct trace back investigations to specific locations.

## Food-borne outbreaks – domestic or non-domestic origin?

Having investigated clusters within the UK that may be geographically restricted, the possibility that isolates of STEC O157:H7 may also exhibit geographical clustering on a global scale, was considered [26]. By tracking the expansion of the three major lineages, the sub-lineages and by superimposing epidemiological data onto the phylogeny e.g. known domestic exposures and recent travel abroad (less than 7 days before onset of symptoms), we can speculate that certain sub-lineages, clades, or clusters may be domestic or non-domestic. For example, sub-lineages Ic and IIb and certain clades and clusters in sub-lineages IIc and I/II are associated with UK strains, whereas sub-lineages 1a, 1b and IIa are likely to be imported from outside the UK. Strains belonging to domestic lineages were more common than non-domestic strains in the routine surveillance collection, and exhibited less diversity within clades because sampling of the restricted pool of diversity (i.e. mainly UK cattle and sheep) was more frequent (Table 2). Included in the domestic clusters, were isolates from UK farm animals [7,24,25,27] and from cases associated with food-borne outbreaks where the food was identified as being of UK origin [7,22,24,28-30] (Table 1).

**Table 2:** Characteristics of domestic and non-domestic clusters of STEC O157:H7, England, 2013–2017

Domestic lineage, clade or cluster Sub-lineages Ic and IIb and clusters within sub-lineages IIc and I/II	Non-domestic lineage, clade Sub-lineages Ia, Ib and IIa and I/II
**Characteristics**	
Common in domestic dataset	Rare in domestic dataset
Short branch lengths (low level diversity) between clusters and clades representing frequent sampling of a restricted pool	Longer branch lengths (high level diversity) between clusters and clades representing infrequent sampling of the global pool
High frequency of domestic animal isolates sampled during prevalence studies and sequenced isolates are included in the dataset	Domestic animal isolates not present
Cases do not report recent travel outside the UK before onset of symptoms	High frequency of cases reporting recent travel before onset of symptoms
Cases from outbreaks known to be associated with domestically produced food,	Cases from outbreaks known to be associated with imported food, Outbreak Scenario 2
Cases associated with local environmental exposures, such as petting farms or parks	Cases not associated with local environmental exposures

STEC: Shiga toxin-producing *Escherichia coli*; UK: United Kingdom.

In contrast, non-domestic clades were more likely to be rare in the UK STEC O157:H7 surveillance database and associated with higher genetic diversity between isolates within a phylogenetic group; representing sparse sampling of a larger pool of diversity (i.e. a wide variety of zoonotic sources dispersed globally) (Table 2). Within these clades, no UK animal isolates were present, as the zoonotic source was located elsewhere, and a high proportion of isolates were from cases reporting foreign travel within 7 days of onset of symptoms (Table 2) [31]. Furthermore, the cases not reporting travel were linked to outbreaks associated with the consumption of imported herbs or salad leaves, or salad leaves grown in the UK from imported seed [28,31,32] (Table 1).

**Figure 2:**
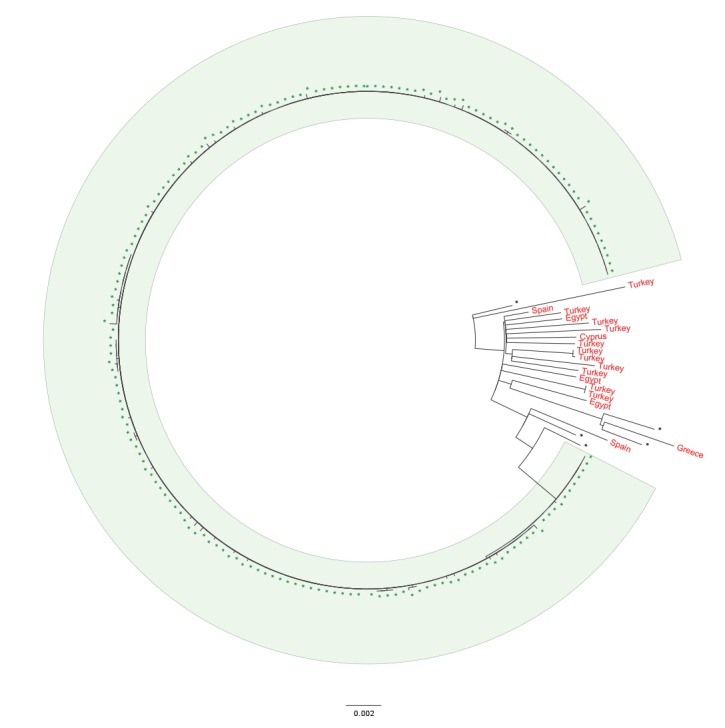
Phylogenetic relationship between isolates associated with an outbreak of red Batavia salad leaves and those from resident cases reporting recent travel to countries in the Mediterranean region, United Kingdom, 2016

Analysis of WGS data from an outbreak in 2016, linked to the consumption of contaminated mixed leaf salad, revealed that the outbreak strain belonged to an uncommon clade in the PHE database and exhibited low levels of sampled diversity, characterised by longer branch lengths indicative of infrequent sampling from a widespread pool of strains [31]. The clade included a high proportion of cases reporting recent travel to Mediterranean countries, compared with other clades in the PHE database (Figure 2). Contaminated imported red Batavia lettuce leaves were suspected as the vehicle of infection, based on the exposure window assessment and supply chain timelines, although no microbiological evidence was obtained [31].

UK: United Kingdom.

Isolates associated with Batavia salad leaves are highlighted in green. Isolates from cases resident in the UK reporting recent travel to countries in the Mediterranean region are highlighted in red.

Quality trimmed Illumina reads were mapped to the STEC O157 reference genome Sakai (Genbank accession BA000007) using BWA-MEM. SNPs were identified using GATK2 in unified genotyper mode. Core genome positions that had a high quality SNP (>90% consensus, minimum depth 10x, MQ ≥ 30) in at least one isolate were extracted. SNP positions that were present in at least 80% of isolates were used to derive maximum likelihood phylogenies with RaxML using the GTRCAT model with 1,000 iterations.

As more countries implement standardised, open access WGS data for routine surveillance of STEC, cross border exchange of WGS data will have a major impact on the ability to investigate national and international outbreaks of food-borne disease [33,34].

## Conclusions

This perspective providing an overview of the use of WGS data during food-borne outbreak investigations in the United Kingdom demonstrated a number of advantages of using this approach: (i) unprecedented level of strain discrimination; (ii) robust, stable genetic markers; (iii) case identification when epidemiological links are obscured; (iv) geographical origins of outbreak strains may be inferred from the phylogenetic signal; and (v) insight into the evolutionary context for emerging pathogenic strains.

We found that collecting detailed epidemiological data is essential to best interpret phylogenetic clusters and that by defining clusters by the number of SNP differences between isolates provides information on strain relatedness. The central tenet of WGS based typing is that the fewer nucleotide differences between a pair of isolates the less time since divergence from a common ancestor i.e. isolates are more likely to originate from the same source population. The amount of diversity sampled when analysing a source population is dependent on the effective size of the population and the duration of infection. Therefore, it is not prudent to define absolute thresholds of nucleotide difference for inclusion and exclusion of isolates within an outbreak and epidemiological information should always be used, where possible, to inform the outbreak definition.

Expanding the use of WGS based typing analysis globally will improve trace back investigations in the event of a food-borne outbreak, ensuring the rapid implementation of interventions to protect public health. For the purposes of risk assessment and management of food-borne outbreaks, the utility of publicly available WGS database linked to the clinical, epidemiological and environmental context of each strain cannot be underestimated.
